# 3-(6-Amino­pyridinium-3-yl)benzoate monohydrate

**DOI:** 10.1107/S1600536812041943

**Published:** 2012-10-13

**Authors:** Zong-Yong Yuan, Jun Zhao, Zhao Peng

**Affiliations:** a College of Mechanical and Material Engineering, China Three Gorges University, Yichang 443002, People’s Republic of China

## Abstract

The title compound, C_12_H_10_N_2_O_2_·H_2_O, crystallizes as a zwitterion in which the pyridine N atom is protonated and the carboxyl OH group is deprotonated. The benzene and pyridinium rings are inclined at a dihedral angle of 54.93 (1)°. In the crystal, O—H⋯O and N—H⋯O hydrogen bonds link the mol­ecules into a three-dimensional supra­molecular network.

## Related literature

For the use of pyridine­carb­oxy­lic acid in coordination chemistry and for related structures, see: Tang *et al.* (2011 ▶); Zhong *et al.* (2008 ▶). 
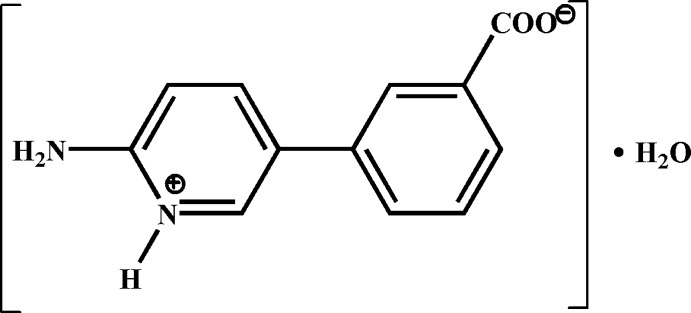


## Experimental

### 

#### Crystal data

C_12_H_10_N_2_O_2_·H_2_O
*M* *_r_* = 232.24
Monoclinic, 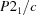

*a* = 7.1956 (18) Å
*b* = 13.091 (9) Å
*c* = 11.987 (10) Å
β = 101.44 (3)°
*V* = 1106.8 (12) Å^3^
*Z* = 4
Mo *K*α radiation
μ = 0.10 mm^−1^
*T* = 296 K
0.20 × 0.18 × 0.17 mm

#### Data collection

Bruker SMART CCD diffractometer
Absorption correction: multi-scan (*SADABS*; Sheldrick, 1996 ▶) *T*_min_ = 0.980, *T*_max_ = 0.983
9294 measured reflections
1942 independent reflections
1344 reflections with *I* > 2σ(*I*)
*R*_int_ = 0.095

#### Refinement

*R*[*F* ^2^ > 2σ(*F* ^2^)] = 0.084
*wR*(*F* ^2^) = 0.215
*S* = 1.09
1942 reflections
154 parameters
H-atom parameters constrained
Δρ_max_ = 0.30 e Å^−3^
Δρ_min_ = −0.21 e Å^−3^

### 

Data collection: *SMART* (Bruker, 1999 ▶); cell refinement: *SAINT* (Bruker,1999 ▶); data reduction: *SAINT*); program(s) used to solve structure: *SHELXS97* (Sheldrick, 2008 ▶); program(s) used to refine structure: *SHELXL97* (Sheldrick, 2008 ▶); molecular graphics: *SHELXTL* (Sheldrick, 2008 ▶); software used to prepare material for publication: *SHELXTL*.

## Supplementary Material

Crystal structure: contains datablock(s) I, global. DOI: 10.1107/S1600536812041943/jj2152sup1.cif

Structure factors: contains datablock(s) I. DOI: 10.1107/S1600536812041943/jj2152Isup2.hkl

Additional supplementary materials:  crystallographic information; 3D view; checkCIF report

## Figures and Tables

**Table 1:** Hydrogen-bond geometry (Å, °)

*D*—H⋯*A*	*D*—H	H⋯*A*	*D*⋯*A*	*D*—H⋯*A*
N1—H1*A*⋯O1^i^	0.86	1.87	2.715 (4)	167
N2—H2*A*⋯O2^i^	0.86	1.95	2.803 (4)	172
N2—H2*B*⋯O1*W*	0.86	2.19	2.915 (5)	142
O1*W*—H1*WA*⋯O2^ii^	0.86	2.00	2.761 (5)	147
O1*W*—H1*WB*⋯O1^iii^	0.87	2.16	2.928 (5)	146

Symmetry codes: (i) 
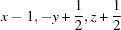
; (ii) 
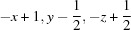
; (iii) 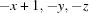.

**Fig. 1.**
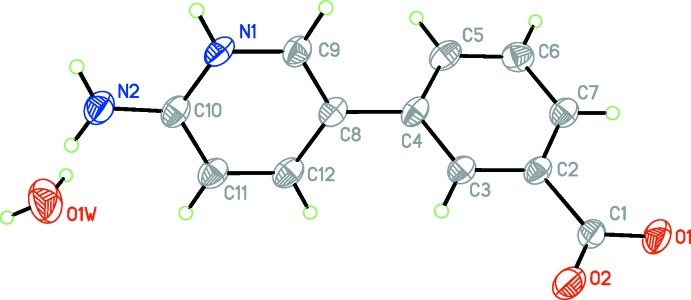
The structure of the title compound with the atom-numbering scheme showing displacement ellipsoids at the 30% probability level for non-H atoms.

**Fig. 2.**
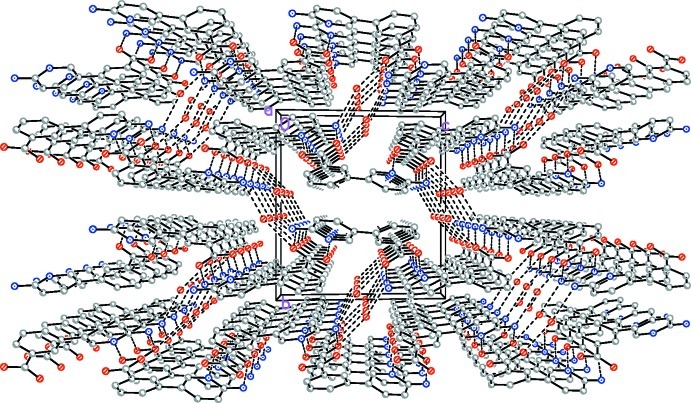
The three-dimensional supramolecular network formed by N—H···O and O—H···O hydrogen-bonding interactions. H atoms not involved in hydrogen bonding have been removed for clarity.

## supplementary crystallographic information

### Comment

Multidentate bridging ligands containing functional groups such as the familiar pyridyl and/or carboxylate groups have proven to be among the most important types of organic ligands for the design and construction of coordination polymers exhibiting remarkable polymeric structural motifs due to their rich coordination modes (Tang *et al.*, 2011; Zhong *et al.*, 2008). We attempted to synthesize a Zn^II^ complex with the ligand in hydrothermal synthesis conditions. However the title compound was obtained, its structure is reported here.

The asymmetric unit of the title compound, C_12_H_10_N_2_O_2_. H_2_O is composed of one 3-(6-Amino-pyridinium-3-yl)-benzoate acid molecule and one lattice water molecule. The dihedral angle between the mean planes of the benzene and pyridinium rings is 54.93 (1)°. The deprotonated carboxylate COO(O1—C1—O2) group is slightly twisted from the benzene ring by an angle of 11.61 (7)° between their mean planes (Fig. 1). Intermolecular O—H···O and N—H···O hydrogen-bonding interactions (Table 1) link adjacent molecules into a three-dimensional supramolecular network (Fig. 2).

### Experimental

A mixture of 3-(6-Amino-pyridin-3-yl)-benzoic acid (0.0214 g, 0.1 mmol), Zn(CH_3_COO)_2_.2H_2_O (0.0219 g, 0.1 mmol) and water (8 ml) was stired vigorously for 30 min and then sealed in a Teflon-lined stainless-steel autoclave. The autoclave was heated and maintained at 393 K for 2 days, and then cooled to room temperature at 5 K h^-1^ to obtain colorless prism crystals suitable for X-ray analysis.

### Refinement

The H atoms bonded to C and N atoms were positioned geometrically (C—H = 0.93 Å, N—H = 0.86 Å) and allowed to ride on their parent atoms, with *U*_iso_(H) value equal to 1.2*U*_eq_(C or N). The H atoms bonded to water O atoms were included in calculated positions and refined with *U*_iso_(H) = 1.5*U*_eq_(O).

### Crystal data

**Table:**

C_12_H_10_N_2_O_2_·H_2_O	*F*(000) = 488
*M**_r_* = 232.24	*D*_x_ = 1.394 Mg m^−^^3^
Monoclinic, *P*2_1_/*c*	Mo *K*α radiation, λ = 0.71073 Å
Hall symbol: -P 2ybc	Cell parameters from 1519 reflections
*a* = 7.1956 (18) Å	θ = 3.1–25.0°
*b* = 13.091 (9) Å	µ = 0.10 mm^−^^1^
*c* = 11.987 (10) Å	*T* = 296 K
β = 101.44 (3)°	Prism, colourless
*V* = 1106.8 (12) Å^3^	0.20 × 0.18 × 0.17 mm
*Z* = 4	

### Data collection

**Table:**

Bruker SMART CCD diffractometer	1942 independent reflections
Radiation source: fine-focus sealed tube	1344 reflections with *I* > 2σ(*I*)
Graphite monochromator	*R*_int_ = 0.095
φ and ω scans	θ_max_ = 25.0°, θ_min_ = 3.1°
Absorption correction: multi-scan (*SADABS*; Sheldrick, 1996)	*h* = −8→8
*T*_min_ = 0.980, *T*_max_ = 0.983	*k* = −15→15
9294 measured reflections	*l* = −14→14

### Refinement

**Table:**

Refinement on *F*^2^	Primary atom site location: structure-invariant direct methods
Least-squares matrix: full	Secondary atom site location: difference Fourier map
*R*[*F*^2^ > 2σ(*F*^2^)] = 0.084	Hydrogen site location: inferred from neighbouring sites
*wR*(*F*^2^) = 0.215	H-atom parameters constrained
*S* = 1.09	*w* = 1/[σ^2^(*F*_o_^2^) + (0.0884*P*)^2^ + 0.8024*P*] where *P* = (*F*_o_^2^ + 2*F*_c_^2^)/3
1942 reflections	(Δ/σ)_max_ < 0.001
154 parameters	Δρ_max_ = 0.30 e Å^−^^3^
0 restraints	Δρ_min_ = −0.21 e Å^−^^3^

### Special details

**Table:**

Geometry. All e.s.d.'s (except the e.s.d. in the dihedral angle between two l.s. planes) are estimated using the full covariance matrix. The cell e.s.d.'s are taken into account individually in the estimation of e.s.d.'s in distances, angles and torsion angles; correlations between e.s.d.'s in cell parameters are only used when they are defined by crystal symmetry. An approximate (isotropic) treatment of cell e.s.d.'s is used for estimating e.s.d.'s involving l.s. planes.
Refinement. Refinement of *F*^2^ against ALL reflections. The weighted *R*-factor *wR* and goodness of fit *S* are based on *F*^2^, conventional *R*-factors *R* are based on *F*, with *F* set to zero for negative *F*^2^. The threshold expression of *F*^2^ > σ(*F*^2^) is used only for calculating *R*-factors(gt) *etc*. and is not relevant to the choice of reflections for refinement. *R*-factors based on *F*^2^ are statistically about twice as large as those based on *F*, and *R*- factors based on ALL data will be even larger.

### Fractional atomic coordinates and isotropic or equivalent isotropic displacement parameters (Å^2^)

**Table:**

	*x*	*y*	*z*	*U*_iso_*/*U*_eq_	
C1	0.6726 (5)	0.2413 (3)	−0.2146 (3)	0.0454 (10)	
C2	0.4946 (5)	0.1869 (3)	−0.1993 (3)	0.0408 (9)	
C3	0.4506 (5)	0.1783 (3)	−0.0921 (3)	0.0418 (9)	
H3A	0.5360	0.2026	−0.0291	0.050*	
C4	0.2823 (5)	0.1342 (3)	−0.0766 (3)	0.0422 (9)	
C5	0.1555 (6)	0.0988 (3)	−0.1711 (4)	0.0518 (11)	
H5A	0.0407	0.0704	−0.1626	0.062*	
C6	0.1995 (6)	0.1055 (3)	−0.2783 (4)	0.0546 (11)	
H6A	0.1146	0.0805	−0.3410	0.066*	
C7	0.3682 (5)	0.1489 (3)	−0.2930 (3)	0.0475 (10)	
H7A	0.3969	0.1526	−0.3652	0.057*	
C8	0.2383 (5)	0.1250 (3)	0.0394 (3)	0.0414 (9)	
C9	0.0750 (5)	0.1644 (3)	0.0640 (3)	0.0461 (10)	
H9A	−0.0115	0.1964	0.0067	0.055*	
C10	0.1542 (5)	0.1128 (3)	0.2573 (3)	0.0442 (10)	
C11	0.3236 (5)	0.0713 (3)	0.2355 (3)	0.0482 (10)	
H11A	0.4080	0.0389	0.2936	0.058*	
C12	0.3642 (5)	0.0783 (3)	0.1300 (3)	0.0478 (10)	
H12A	0.4779	0.0517	0.1172	0.057*	
N1	0.0361 (4)	0.1579 (2)	0.1700 (3)	0.0454 (8)	
H1A	−0.0681	0.1837	0.1819	0.054*	
N2	0.1056 (5)	0.1100 (3)	0.3583 (3)	0.0558 (10)	
H2A	0.0003	0.1366	0.3671	0.067*	
H2B	0.1795	0.0814	0.4149	0.067*	
O1	0.7197 (4)	0.2360 (2)	−0.3109 (2)	0.0626 (9)	
O1W	0.2996 (5)	−0.0676 (3)	0.4736 (3)	0.0984 (13)	
H1WA	0.3198	−0.0995	0.5372	0.148*	
H1WB	0.2433	−0.1120	0.4237	0.148*	
O2	0.7649 (4)	0.2900 (2)	−0.1316 (2)	0.0611 (9)	

### Atomic displacement parameters (Å^2^)

**Table:**

	*U*^11^	*U*^22^	*U*^33^	*U*^12^	*U*^13^	*U*^23^
C1	0.040 (2)	0.050 (2)	0.048 (2)	0.0020 (18)	0.0135 (18)	0.007 (2)
C2	0.0366 (19)	0.038 (2)	0.052 (2)	0.0037 (16)	0.0172 (17)	0.0019 (17)
C3	0.039 (2)	0.043 (2)	0.046 (2)	0.0025 (17)	0.0136 (17)	−0.0019 (17)
C4	0.043 (2)	0.034 (2)	0.055 (2)	−0.0038 (16)	0.0223 (18)	−0.0001 (17)
C5	0.045 (2)	0.046 (2)	0.068 (3)	−0.0120 (19)	0.019 (2)	−0.007 (2)
C6	0.051 (3)	0.055 (3)	0.055 (3)	−0.008 (2)	0.004 (2)	−0.007 (2)
C7	0.047 (2)	0.045 (2)	0.053 (2)	0.0009 (19)	0.0162 (19)	−0.0021 (19)
C8	0.043 (2)	0.0304 (19)	0.055 (2)	0.0031 (16)	0.0192 (18)	0.0039 (17)
C9	0.046 (2)	0.043 (2)	0.053 (2)	−0.0009 (18)	0.0183 (19)	0.0058 (18)
C10	0.047 (2)	0.035 (2)	0.056 (2)	−0.0028 (17)	0.0215 (19)	0.0021 (18)
C11	0.046 (2)	0.043 (2)	0.060 (3)	0.0058 (18)	0.0190 (19)	0.0039 (19)
C12	0.044 (2)	0.039 (2)	0.065 (3)	0.0067 (18)	0.023 (2)	−0.0010 (19)
N1	0.0381 (17)	0.0437 (18)	0.060 (2)	0.0043 (15)	0.0242 (16)	0.0041 (16)
N2	0.053 (2)	0.062 (2)	0.058 (2)	0.0078 (17)	0.0229 (17)	0.0039 (17)
O1	0.0569 (18)	0.089 (2)	0.0477 (17)	−0.0125 (16)	0.0258 (14)	−0.0030 (15)
O1W	0.120 (3)	0.107 (3)	0.070 (2)	0.030 (3)	0.024 (2)	0.032 (2)
O2	0.0484 (16)	0.083 (2)	0.0543 (18)	−0.0193 (16)	0.0175 (14)	−0.0085 (16)

### Geometric parameters (Å, º)

**Table:**

C1—O2	1.256 (4)	C8—C12	1.408 (5)
C1—O1	1.267 (5)	C9—N1	1.357 (5)
C1—C2	1.508 (5)	C9—H9A	0.9300
C2—C3	1.387 (5)	C10—N2	1.326 (5)
C2—C7	1.390 (5)	C10—N1	1.346 (5)
C3—C4	1.387 (5)	C10—C11	1.406 (5)
C3—H3A	0.9300	C11—C12	1.357 (5)
C4—C5	1.386 (5)	C11—H11A	0.9300
C4—C8	1.491 (5)	C12—H12A	0.9300
C5—C6	1.385 (5)	N1—H1A	0.8600
C5—H5A	0.9300	N2—H2A	0.8600
C6—C7	1.383 (5)	N2—H2B	0.8600
C6—H6A	0.9300	O1W—H1WA	0.8554
C7—H7A	0.9300	O1W—H1WB	0.8736
C8—C9	1.368 (5)		
			
O2—C1—O1	123.7 (4)	C9—C8—C4	121.4 (4)
O2—C1—C2	118.1 (3)	C12—C8—C4	122.1 (3)
O1—C1—C2	118.2 (4)	N1—C9—C8	121.6 (4)
C3—C2—C7	119.1 (3)	N1—C9—H9A	119.2
C3—C2—C1	120.5 (3)	C8—C9—H9A	119.2
C7—C2—C1	120.3 (3)	N2—C10—N1	118.8 (3)
C4—C3—C2	121.5 (4)	N2—C10—C11	123.6 (4)
C4—C3—H3A	119.2	N1—C10—C11	117.6 (3)
C2—C3—H3A	119.2	C12—C11—C10	120.1 (4)
C5—C4—C3	118.7 (4)	C12—C11—H11A	120.0
C5—C4—C8	120.6 (3)	C10—C11—H11A	120.0
C3—C4—C8	120.7 (4)	C11—C12—C8	121.6 (4)
C6—C5—C4	120.2 (4)	C11—C12—H12A	119.2
C6—C5—H5A	119.9	C8—C12—H12A	119.2
C4—C5—H5A	119.9	C10—N1—C9	122.7 (3)
C7—C6—C5	120.8 (4)	C10—N1—H1A	118.7
C7—C6—H6A	119.6	C9—N1—H1A	118.7
C5—C6—H6A	119.6	C10—N2—H2A	120.0
C6—C7—C2	119.6 (4)	C10—N2—H2B	120.0
C6—C7—H7A	120.2	H2A—N2—H2B	120.0
C2—C7—H7A	120.2	H1WA—O1W—H1WB	105.1
C9—C8—C12	116.5 (3)		
			
O2—C1—C2—C3	−9.5 (5)	C5—C4—C8—C9	−55.5 (5)
O1—C1—C2—C3	171.0 (4)	C3—C4—C8—C9	124.6 (4)
O2—C1—C2—C7	167.5 (4)	C5—C4—C8—C12	126.5 (4)
O1—C1—C2—C7	−11.9 (5)	C3—C4—C8—C12	−53.5 (5)
C7—C2—C3—C4	−1.1 (5)	C12—C8—C9—N1	−0.6 (6)
C1—C2—C3—C4	176.0 (3)	C4—C8—C9—N1	−178.7 (3)
C2—C3—C4—C5	−0.4 (5)	N2—C10—C11—C12	−179.2 (4)
C2—C3—C4—C8	179.6 (3)	N1—C10—C11—C12	0.7 (6)
C3—C4—C5—C6	1.5 (6)	C10—C11—C12—C8	−1.3 (6)
C8—C4—C5—C6	−178.5 (4)	C9—C8—C12—C11	1.2 (6)
C4—C5—C6—C7	−1.1 (6)	C4—C8—C12—C11	179.3 (4)
C5—C6—C7—C2	−0.5 (6)	N2—C10—N1—C9	179.9 (4)
C3—C2—C7—C6	1.5 (5)	C11—C10—N1—C9	−0.1 (5)
C1—C2—C7—C6	−175.6 (3)	C8—C9—N1—C10	0.0 (6)

### Hydrogen-bond geometry (Å, º)

**Table:**

*D*—H···*A*	*D*—H	H···*A*	*D*···*A*	*D*—H···*A*
N1—H1*A*···O1^i^	0.86	1.87	2.715 (4)	167
N2—H2*A*···O2^i^	0.86	1.95	2.803 (4)	172
N2—H2*B*···O1*W*	0.86	2.19	2.915 (5)	142
O1*W*—H1*WA*···O2^ii^	0.86	2.00	2.761 (5)	147
O1*W*—H1*WB*···O1^iii^	0.87	2.16	2.928 (5)	146

Symmetry codes: (i) *x*−1, −*y*+1/2, *z*+1/2; (ii) −*x*+1, *y*−1/2, −*z*+1/2; (iii) −*x*+1, −*y*, −*z*.
